# Debunking the Myth of Value-Neutral Virginity: Toward Truth in Scientific Advertising

**DOI:** 10.3389/fpsyg.2016.00451

**Published:** 2016-03-30

**Authors:** David R. Mandel, Philip E. Tetlock

**Affiliations:** ^1^Socio-Cognitive Systems Section, Defence Research and Development Canada and Department of Psychology, York UniversityToronto, ON, Canada; ^2^Wharton School and the School of the Arts and Sciences, University of PennsylvaniaPhiladelphia, PA, USA

**Keywords:** value neutrality, scientific behavior, social functionalism, scientific advisors, policymakers

## Abstract

The scientific community often portrays science as a value-neutral enterprise that crisply demarcates facts from personal value judgments. We argue that this depiction is unrealistic and important to correct because science serves an important knowledge generation function in all modern societies. Policymakers often turn to scientists for sound advice, and it is important for the wellbeing of societies that science delivers. Nevertheless, scientists are human beings and human beings find it difficult to separate the epistemic functions of their judgments (accuracy) from the social-economic functions (from career advancement to promoting moral-political causes that “feel self-evidently right”). Drawing on a pluralistic social functionalist framework that identifies five functionalist mindsets—people as intuitive scientists, economists, politicians, prosecutors, and theologians—we consider how these mindsets are likely to be expressed in the conduct of scientists. We also explore how the context of policymaker advising is likely to activate or de-activate scientists’ social functionalist mindsets. For instance, opportunities to advise policymakers can tempt scientists to promote their ideological beliefs and values, even if advising also brings with it additional accountability pressures. We end prescriptively with an appeal to scientists to be more circumspect in characterizing their objectivity and honesty and to reject idealized representations of scientific behavior that inaccurately portray scientists as value-neutral virgins.

Policymakers seek scientific advice for a plurality of reasons. Sometimes, they seek accurate, actionable information to guide their decisions, and call on scientists to act as honest information brokers. At other times, policymakers might view advisors more as influence tools than information brokers, wagering that the “right scientists" could help sway public opinion in favor of preferred policy stances on controversial issues. To that end, they will seek advisors who have cachet inside the scientific community and who share their ideological agenda. In most cases, however, policymakers have mixed motives. They want to be honestly informed, but are ready to use the tactics of motivated reasoning if presented with evidence that challenges their ideological frameworks and preferred policy stances (Kunda, 1990). They may not be so ideologically committed as to ignore overwhelming contradictory evidence, but neither are they likely to engage advisors with neutral priors. In short, policymakers face value tradeoffs when they seek scientific advice.

In this essay, we examine the other side of the equation—scientists as advisors, and in their everyday roles as researchers. Our thesis, however, resembles our opening statement about policymakers—namely, scientists’ motives are characterized by value tradeoffs that shape their behavior. Yet, whereas it may come as no surprise that policymakers may push their policy agendas forward even if it means sacrificing some truth, there is much cultural lore militating against this ascription to scientists. We contend that attempts to hold the scientific community to a pristine standard of value neutrality ring hollow upon closer inspection. As scientists, we should aim to be objective about the fact that none of us is 100% objective—and indeed honest about the fact that none of us is 100% honest. We do not offer this viewpoint as an exculpatory thesis for scientific wrongdoings. Nor do we wish to diminish the importance of scientists’ advisory roles, which we regard as of significant real and potential value. Yet, we argue that a healthy dose of truth in scientific advertising would help resolve glaring inconsistencies in scientific conduct that challenge the coherence of the value-neutral narrative.

## Debunking The Myth Of Value Neutrality

What can a truth-seeking policymaker realistically expect from scientific advisors? If we take the scientific community at its word, the short answer is a lot. The scientific community presents its members as dispassionate, value-neutral enterprisers dedicated to advancing knowledge and to clearly demarcating where the facts end and speculation begins (Mulkay, 1979; Gieryn, 1983, 1999). Science is portrayed as above the political fray—and scientists as non-partisan truth-seekers who know how to separate their factual judgments from their value judgments—and who are committed to doing so.

Scientists might concede that there is nothing wrong with using value judgments to guide the *application* of science, including personal decisions regarding when and how to assist policymakers. Some might even argue that it would be ethically irresponsible to try to duck such judgments. However, most scientists believe that once an area of application is chosen, the scientific process and the information gleaned from it should be unaffected by personal values. Ideally, in this view, the “doing” of science—from generating hypotheses to designing research to evaluating hypotheses—should be value-neutral and conform to Merton’s (1942) canonical CUDOS norms of science; namely, Communism (openness and sharing of ideas and data), Universality (inclusiveness and rejection of evaluating work of other scientists on ideological or ethnic-racial grounds), Disinterestedness (applying the same standards of evidence and proof to one’s own theories and to rival theories), and Organized Skepticism (subjecting all scientific claims, especially one’s own, to critical scrutiny of peer review).

Scientists and policymakers alike surely realize there are exceptions to the CUDOS norms, as when scientists are caught having fabricated data. The scientific community’s reaction to such cases, which couples surprise, outrage and scorn, suggests that blatant misconduct is merely the work of a few bad apples—flawed characters who never internalized scientists’ professional code of conduct. Yet evidence suggests otherwise. For instance, a meta-analysis of studies examining unethical research practices found that, on average, 2% of scientists admitted to having personally committed egregious forms of scientific misconduct—falsification, fabrication, or modification of data—in their research, and 14% claimed they had observed other researchers doing so (Fanelli, 2009). These figures are surely conservative given strong incentives not to report misconduct, especially one’s own. Barring extreme self-deception, it should be more difficult to detect such misconduct in others’ research than in one’s own. Thus, one might infer that 14% is close to a minimum rate of the most serious forms of misconduct. A rate so high simply cannot be meshed with the “few bad apples” dispositional narrative. It should prompt policymakers and the public to question the extent to which the advice they are receiving from scientists is sound. And it should prompt the scientific community to seek better explanations of scientific behavior, including misconduct.

Although findings of misconduct and of the widespread nature of improper methodological practices are now well publicized (e.g., Ioannidis, 2005; Simmons et al., 2011), there remains a need for a theoretical framework within which these findings can be better understood. Without denying the value of CUDOS norms, we question whether such a normative framework–or indeed whether any framework that might defensibly be called normative—can serve as an adequate *descriptive* account of scientific behavior. We argue that the normative-descriptive gap is wider than most scientists care to admit or realize, and that a plausible descriptive account of scientific behavior is needed.

## Toward a Pluralistic Social Functionalist Account of Scientific Behavior

In sketching the outline of such an account, we draw on Tetlock’s (2002) social functionalist framework, which stresses the plurality of goals driving human behavior (see also Kunda, 1990; Alicke et al., 2015). The framework acknowledges that two functionalist metaphors have dominated the study of judgment and choice: people as intuitive scientists and as intuitive economists. The former posits the quest for truth to be a central goal guiding human activity, whereas the latter posits that goal to be utility maximization. Each metaphor has proven useful in stimulating dynamic research programs in social science (e.g., attribution theories in the former case and rational choice theories in the latter case).

Nevertheless, the framework posits a need for an expanded repertoire of social functionalist metaphors that permit individuals to be described in pluralistic terms that capture their central goals across a wide range of social contexts posing different adaptive challenges. In particular, Tetlock (2002) proposed three additional metaphors: people as intuitive politicians, prosecutors, and theologians. The intuitive politician mindset is triggered when individuals experience accountability pressure from important audiences. Such pressures trigger the goal of maintaining a favorable social identity or promoting one’s reputation to the relevant audiences. That goal, in turn, triggers a range of behavioral strategies, such as pre-emptive self-criticism or defensive bolstering, which are contingent on the relation between the intuitive politician and his audience (Lerner and Tetlock, 1999). In contrast, the intuitive prosecutor mindset is prompted by the observer’s perception that societal norm violators are plentiful and frequently go unpunished (Tetlock et al., 2007). Whereas the intuitive politician responds to accountability pressures by opening loopholes that increase moral wiggle room, the intuitive prosecutor seeks to intensify such pressures on others that close loopholes. For instance, subjects assigned more blame to a cheater whose cheating behavior caused a non-cheater to suffer a loss when cheating was normative (i.e., many cheaters) than when cheating was counter-normative (Alicke et al., 2011). The social functional framework predicts that common social-norm violations should trigger more extreme prosecutorial responses than occasional violations because threats to control are more severe in the former case. Finally, the intuitive-theologian mindset gives intuitive prosecutors backbone: the prosecutorial mission is not just to enforce social conventions but rather to protect foundational community values—science’s *sacred values* (Tetlock et al., 2000)—against secular encroachments, like scientists being tempted to fake data for financial gain or worldly fame. An important characteristic of the intuitive-theologian mindset is its resistance to tradeoffs that in any way compromise sacred values. For instance, people are much more likely to deny that some benefit might be accrued by violating sacred values compared to non-sacred values they merely oppose (Baron and Spranca, 1997).

Any adequate description of scientific behavior requires a pluralistic brand of social functionalism because scientists, like ordinary mortals, must balance cross-pressures and competing goals. Pluralistic social functionalism offers a range of metaphors sufficient to encode the goals, value tradeoffs, and behavioral responses of actors and observers that arise within scientific communities, bearing in mind that scientists will exhibit individual differences in their goals and how they resolve goal or value conflicts. It is thus useful to consider scientists from the perspective of each of the five metaphorical mindsets. The obvious starting point is the intuitive scientist who, as noted earlier, is motivated by purely epistemic goals. This is the scientist as the ideal Weberian type (Weber, 1904/1949, 1917/1949)—unwilling to inject value judgments into scientific practice and, as an advisor, seeking only to use science to elucidate the most effective means of realizing the policymaker’s stated goals.

We may juxtapose that view against the scientist as intuitive economist. Today’s scientists were once students who made career choices among a range of feasible options given their interests, aptitudes, and opportunities. As in any profession, members quickly learn the profession’s incentive structures and take steps to advance their material, reputational, and even ideological interests within the ground rules. Therefore, as intuitive economists, scientists are poised to engage a repertoire of goal-advancement tactics, including the exploitation of loopholes within their profession, which allow them to realize their multiple self-interests. For instance, although scientists become aware of the CUDOS norms (at least in spirit) early in their careers, they might opt (or be advised by mentors) to disregard the norm of Communism in favor of holding career-advancing ideas or findings close to the chest until they are published.

It is impossible, however, to obtain an accurate view of scientists’ behavior without applying the mindset metaphors interactively. For example, consider the mental calculus of scientists as intuitive economists. In deciding how to advance their interests, they must assess the likely reactions of colleagues from the perspective of the intuitive politician. As members of a professional community, scientists cannot ignore these accountability pressures without consequences. We suspect that a careful analysis of the tensions between the intuitive economist and intuitive politician mindsets would help to explain the frequency distribution of misdemeanor types in science. That is, when the intuitive politician judges the reputational risks of the intuitive economist’s pragmatic tactics to be low, we expect a community-wide spike in such activity. Types of normative infractions that are consensually ignored by community members—the equivalent of jaywalking in any large North American city—and which thus carry low anticipatory accountability costs should be frequently observed with little concealment effort. A scientist might be quite open about not wanting to share exciting new findings before they are published, while being unwilling to disclose the fact that they selectively relegate studies to the proverbial file drawer. Yet, where the scientific community incentivizes taboo practices, such as selectively reporting results that are likely to entice peer reviewers and editors or torturing the data until a statistically significant finding gives itself up (Simonsohn et al., 2014), we should see a rise in their prevalence, signaling a shift toward cargo-cult science (Feynman, 1974). In fact, selective reporting is more prevalent in scientific contexts that strongly incentivize such practices (Fanelli, 2010, 2012), and where opportunities for data manipulation flourish (Fanelli and Ioannidis, 2013).

The preceding examples foreshadow the need for what might seem the unlikeliest metaphorical contender for modeling scientific behavior: the intuitive-theologian mindset. Science, after all, is supposed to be the antithesis of dogma, and it has over the last four centuries rolled back the authority of theologians to explain the workings of the natural world. Nevertheless, we argue that the scientific community is dogmatically inculcated with a normative value system that, among other things, teaches scientists to believe—or at least act as if they believe—that they are engaged in a value-neutral enterprise. Such beliefs, partly captured by the CUDOS norms, amount to the community’s sacred values, which serve multiple functions. First, and consistent with the scientific community’s self-narrative, such values support truth discoveries as an epistemic priority. Second, they help to unify the scientific community and contribute to a shared sense of purpose or “collective consciousness” as Durkheim (1893/2015) had put it. Third, they validate scientific practices within the broader society and bolster the community’s reputation much as the Hippocratic oath functions in medicine. In effect, the norms serve as part of science’s vocabulary of ideological self-description to the public (Mulkay, 1976), positively differentiating science from other knowledge-generation societies (Gieryn, 1999).

Perhaps foremost among dogmatic claims in science’s “secular theology” is the fact-value dichotomy. The philosophical arguments for the claim that science is fact-laden and value-neutral have been successfully refuted in stages, starting with Quine’s (1951) attack on the dogmas of logical empiricism and ending with Putnam’s (2002) pragmatist attack on the dichotomy itself. Yet, from a descriptive viewpoint, we expect scientists to continue to defend the dogma as an unassailable truth, and we expect scientists to respond predictably to attacks on sacred beliefs. Thus, attacks on the value-neutrality of science are likely to be dismissed as unworthy of response and, if persistent, to draw out sharp counterattacks, like *ad hominem* ridicule and ostracism.

While intellectual attacks on science’s sacred values, we predict, will trigger intuitive-theologian defense mechanisms, self-interested violators of science who are caught doing things that “give science a bad name” will activate their peers’ prosecutorial mindset. As noted earlier, the scientific community responds to norm violators by characterizing them as a few bad apples, thus obfuscating deeper structural problems that incentivize unsanctionable norm violations in the first place. In effect, the scientific community prosecutes members who fail to ensure that their inner intuitive politicians keep their greedy, inner intuitive economists adequately in check.

To sum up, our perspective on scientific behavior is that there is no single, unadulterated “view from nowhere” in science, to use Nagel’s (1986) phrase. Scientists inevitably view their subject matter from multiple, difficult-to-reconcile viewpoints. It would be wrong to conclude, however, that we are trying to eradicate the narrative of scientists as truth seekers. Although we reject a singular idealist narrative along those lines, we likewise reject singular cynical narratives. For instance, we reject portrayals of scientists as mere hucksters peddling their latest epistemic wares. We argue that the challenge for any adequate descriptive account of scientific behavior—and indeed social behavior in any realm—is to resist the simplistic appeal of characterizations that assign victory to a single perspective. Our view is deeply pluralistic in the spirit of Berlin (1990) who, following Kant, warned us to expect no straight thing to be built from the crooked timber of humanity.

## Scientists in the Advisory Context

The advisory context affects the scientist’s social functionalist mindsets, but to varying degrees and in different respects. For instance, the intuitive-scientist mindset will mainly be affected in terms of its “flavor.” As advisors, scientists maintain their epistemic goals, but because policymakers seek practical advice and can care less about theory development (Sunstein, 2015), the scientist’s epistemic focus—at the insistence of the intuitive politician—will be tempered by pragmatism (e.g., timeliness and relevance to the policymaker’s concerns).

In comparison, the intuitive economist’s synapses are likely to fire rapidly in response to opportunities to advise policymakers. Such opportunities can yield extrinsic and intrinsic economic benefits to advisors, such as lucrative consulting fees and status. If the advising context is well matched to the advisor’s ideological commitments, opportunities to influence power-holders’ views on topics of value importance could also send the advisor’s intuitive-theologian into a frenzied state. In such cases, the scientist as intuitive-theologian is faced with balancing commitments to competing sacred values, including those of the scientific community. Unsurprisingly, in such battles, the scientists’ personal values often win out, leading them to adopt questionable interpretative practices favoring their ideological commitments (Jussim et al., 2016). For example, not only is there a prevalent liberal bias in social science, but many social scientists admit they would discriminate against colleagues who do not share their political views (Inbar and Lammers, 2012; Duarte et al., 2015). One of the greatest costs of ritualizing scientific values is that they will not be strongly internalized, as work on value pluralism suggests (Tetlock, 1986).

Within the advisory context, the intuitive politician is bound to work overtime. For academics plucked from their usual roles, accountability pressures of advising policymakers will be less familiar, prompting more effortful consideration of appropriate response strategies. For instance, advisors may need to think through the extent to which they will deliver advice in a fox-like style, with large dollops of preemptive self-criticism, risking appearing cowardly albeit balanced, or deliver advice in a more decisive hedgehog-like style, risking appearing dogmatic albeit decisive (Tetlock, 2005). The intuitive politician, however, faces challenges that go well beyond those posed by the novelty of the audience and which extend to ensuring that the temptations of the intuitive economist and theologian mindsets are reasonably kept in check.

## Where Does this Leave Us?

If the notion of value neutrality in science is a mythical holdover from logical positivism (Putnam, 2002), and if a murky mix of social-functionalist goals actually governs scientific conduct, where does it leave us? In the end, we appeal to scientists to try to be objective about our imperfect objectivity—and honest that none of us is capable of being perfectly honest given the vying mindsets that shape our goals. Such epistemic modesty is more in keeping with the scientific spirit of non-dogmatic inquiry than blind adherence to the sacred narrative of value-neutral virginity. If done right, vanquishing the tenacious myth of value neutrality could make us truer to the values of science and more honest as advisors. Yet, there is a risk that honesty about our non-epistemic goals could be used to condone the very practices that harm scientific integrity. Scientists must walk a fine line.

## Author Contributions

All authors listed, have made substantial, direct and intellectual contribution to the work, and approved it for publication.

## Conflict of Interest Statement

The authors declare that the research was conducted in the absence of any commercial or financial relationships that could be construed as a potential conflict of interest.
